# Supplementary material for: The Effects of Light Spectrum and Intensity, Seeding Density, and Fertilization on Biomass, Morphology, and Resource Use Efficiency in Three Species of Brassicaceae Microgreens
Source: Plants (Basel). 2024 Jan 1;13(1):124. doi: 10.3390/plants13010124 (PMC10780592; doi:10.3390/plants13010124)
Supplement: Supplementary file 1 [file plants-13-00124-s001.zip › plants-2777913-supplementary.pdf]

plants

MDPI

1. Supplementary Materials

Table S1. Mean 100 seed weights (± SE), Standard and High Seeding Density (g/cup), and estimated number of seeds per cup at Standard and High Seeding Density, and mean germination percentage (± SE).

Species	*100 Seed Weight (g)*	*Standard SD (g/cup)*	*Estimated Seeds/cup*	*High SD (g/cup)*	*Estimated Seeds/cup*	*Germ %*
Kohlrabi	0.16 ± 0.01	1.50	930	2.25	1394	91.45 ± 0.36
Mustard	0.72 ± 0.01	3.00	417	4.50	625	99.81 ± 0.03
Radish	1.33 ± 0.02	4.00	302	6.00	453	99.69 ± 0.05

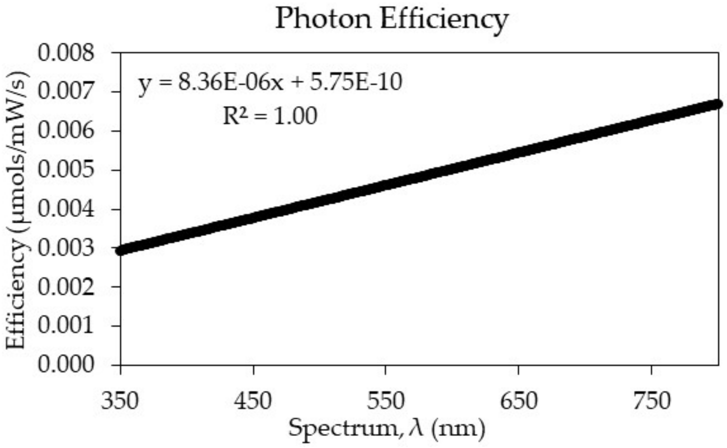

Figure S1. Linear regression for the production ratio of micromoles of photons generated per unit energy output, per second, for wavelengths of light from 350 to 800 nm. Datapoints generated for every nanometer (nm) of light wavelength. Linear equation and R^2 are displayed in upper left.

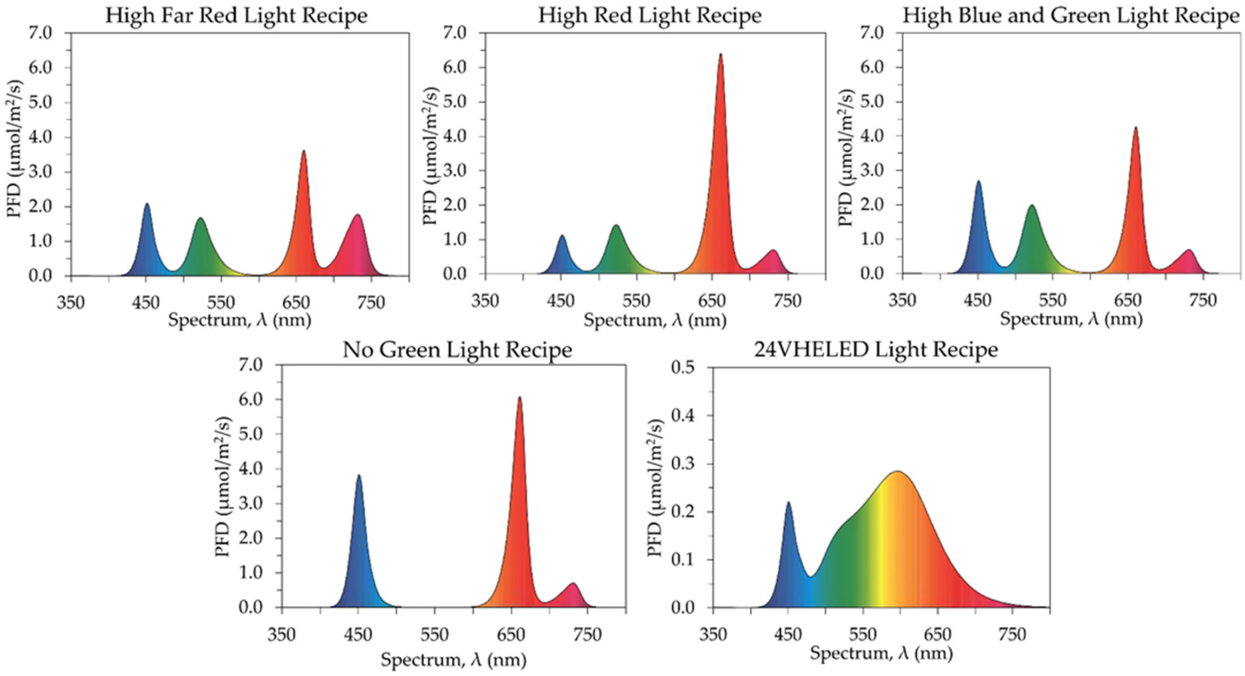

Figure S2. Panels showing the PFD (μmol/m²/s) for each Light Recipe, from upper left to bottom right: High Far-Red, High Red, High Blue and Green, No Green, and 24VHELED. Values generated from spectrometric readings at a resolution of 1 nm.

1.1 Kohlrabi

1.1.1 Kohlrabi Production

The following results are included in this publication as supplementary materials to provide a higher resolution perspective regarding the species-specific responses to our experimental conditions. The multiway ANOVA results from Table S2 demonstrate that for kohlrabi, there were significant differences for Fresh Weight biomass production due to Light Recipe, Fertilizer, and SD main effects ($p < 0.001$, $p < 0.001$, and $p < 0.001$, respectively). There was also significant interaction between Fertilizer and SD ($p < 0.001$). This indicates that the production of fresh biomass is a function of the different Light Recipes, and is modified by the combination of Fertilizer and SD. The large Sum Squares of the Fertilizer and SD model components is not surprising, as more seeds results in more biomass, and applying Fertilizer also improves biomass accumulation greatly. These effects explained more variation than the Light Recipe (31.98% and 47.94% compared to 7.60%, respectively), or any other model effect. For DW, we found very similar results, also shown in Table S2, except that there were no significant higher order interactions. For DW, the main effects for Light Recipe, Fertilizer, and Seeding Density were all highly significant ($p < 0.001$, $p < 0.001$, and $p < 0.001$, respectively). The effect size for Light Recipe was more than double for DW than it was for FW (19.16%), and Fertilizer decreased as well (29.06%), while SD increased (35.76%).

Table S2. Type I ANOVA summary table for kohlrabi microgreen FW (left) and DW (right). Sources of variation are Light Recipe (LR), Fertilizer (Fert.), Seeding Density (SD), and their interactions. The df is degrees of freedom, SS is sum squares, η^2 is eta-squared as the effect size, MS is mean squares, and p is the p-value. Significance set at $\alpha = 0.05$.

Source of Variation	*df*	*SS (FW)*	*η^2 (FW)*	*MS (FW)*	*p (FW)*	*SS (DW)*	*η^2 (DW)*	*MS (DW)*	*p (DW)*
LR	4	1.05	7.60%	0.26	< 0.001	6.38E-03	19.16%	1.60E-03	< 0.001
Fert.	1	6.60	47.94%	6.60	< 0.001	9.68E-03	29.06%	9.68E-03	< 0.001
SD	1	4.40	31.98%	4.40	< 0.001	1.19E-02	35.76%	1.19E-02	< 0.001
LR:Fert.	4	0.04	0.31%	0.01	0.63	5.34E-04	1.60%	1.33E-04	0.07
Fert.:SD	1	0.30	2.16%	0.30	< 0.001	7.90E-05	0.24%	7.90E-05	0.12
LR:SD	4	0.02	0.16%	0.01	0.86	2.50E-04	0.75%	6.24E-05	0.14
LR:Fert.:SD	4	0.11	0.78%	0.03	0.17	4.98E-04	1.50%	1.25E-04	0.23
Error		1.25	9.07%	0.02		3.98E-03	11.95%	5.24E-05	

Because of the expectation that adding Fertilizer and increasing the seeding density would significantly contribute to biomass improvements, we conducted linear model analyses for just Light Recipe to isolate the dynamics in this single main effect. For instance, Table S3 (FW, top) and Table S4 (DW, bottom) expands on the relationship between light components and biomass production by showing the R^2, model estimate coefficient, effect size (η^2), and p-value (p) at each treatment level. The linear models used four LED bands (Blue, 400-500 nm; Green, 500-600 nm; Red, 600-700 nm; and Far-Red, 700-800) as predictive variables for biomass accumulative variation. Overall, Table S3 shows that, for FW, Far-Red light was significantly associated with increased biomass accumulation at all combinations of experimental factors. Interestingly, Blue and Red model components were significantly negatively associated with fresh biomass production for kohlrabi when no Fertilizer was applied. Furthermore, Green light was nearly significantly associated with FW biomass at the Unfertilized and Standard SD treatment combination ($p=0.07$). For DW (Table S4), similar results were seen, with Far-Red being significantly positively associated with all experimental factor combinations. Red light was also positively significantly associated with DW production at all treatment combinations, while Blue was negatively associated. Overall, these linear models, using only light parameters as inputs for predicting

FW and DW biomass, had coefficients of determination (R^2) ranging from 0.36 at the lowest, to a high of 0.80, with an average R^2 of 0.64.

Table S3. Linear model R^2, coefficients, effect size (%), and effect significance (p) for kohlrabi FW at different treatment combinations (Fertilizer, SD, and wavelength band). Wavelength band was defined as Blue (400-500 nm), Green (500-600 nm), Red (600-700 nm), and Far-Red (700-800).

Fertilizer	*SD*	*Effect*	*R^2 of Model*	*Coefficient*	*η^2*	*p*
Yes	Standard	Far-Red	0.50	4.60E-03	15.78%	0.02
Yes	High	Far-Red	0.36	7.06E-03	22.95%	0.01
No	Standard	Green	0.75	1.51E-03	31.54%	0.07
No	Standard	Red	0.75	-9.09E-04	11.88%	< 0.01
No	Standard	Far-Red	0.75	4.71E-03	28.50%	< 0.001
No	High	Blue	0.59	-3.25E-03	7.80%	0.01
No	High	Far-Red	0.59	5.40E-03	22.53%	< 0.01

Table S4. Linear model R^2, coefficients, effect size (%), and effect significance (p) for kohlrabi DW at different treatment combinations (Fertilizer, SD, and wavelength band). Wavelength band was defined as Blue (400-500 nm), Green (500-600 nm), Red (600-700 nm), and Far-Red (700-800).

Fertilizer	*SD*	*Effect*	*R^2 of Model*	*Coefficient*	*η^2*	*p*
Yes	Standard	Red	0.70	1.14E-04	18.61%	< 0.01
Yes	Standard	Far-Red	0.70	3.03E-04	12.22%	< 0.01
Yes	High	Red	0.80	8.71E-05	10.30%	0.04
Yes	High	Far-Red	0.80	6.32E-04	31.10%	< 0.001
No	Standard	Red	0.66	5.61E-05	9.69%	0.06
No	Standard	Far-Red	0.66	2.71E-04	15.38%	0.02
No	High	Blue	0.76	-2.35E-04	0.97%	< 0.01
No	High	Red	0.76	1.46E-04	36.30%	< 0.001
No	High	Far-Red	0.76	3.87E-04	18.40%	< 0.01

The Pearson's Correlation in Figure S3 below shows the correlations between our primary dependent variables of interest, as well as some of our key independent ones, such as light components, ratios, Fertilizer and SD. Red coloration indicates positive correlation, and blue indicates negative correlation; asterisks indicate significance from * = $p < 0.05$, ** = $p < 0.01$, *** = $p < 0.001$. Figure S3 shows that FW was positively, but not significantly, correlated with Green and Far-Red light components. For DW, more light (TPFD) and light components (B, G, R, and FR) were significantly correlated with biomass. DW pct had many significant positive and negative correlations with light treatments; of note is the lack of correlation with Green light. For FW relative production (g FW/g seeding density), Green and Far-Red light were significantly correlated, indicating that there was more relative per plant production with Light Recipes that had higher amounts of Far-Red and Green light, which was what we hypothesized would occur in highly competitive, dense microgreens environments. For relative DW production, it was once again all types of light that were significantly correlated. For our RUE parameters, applying Fertilizer or increasing SD significantly positively correlated with outcomes.

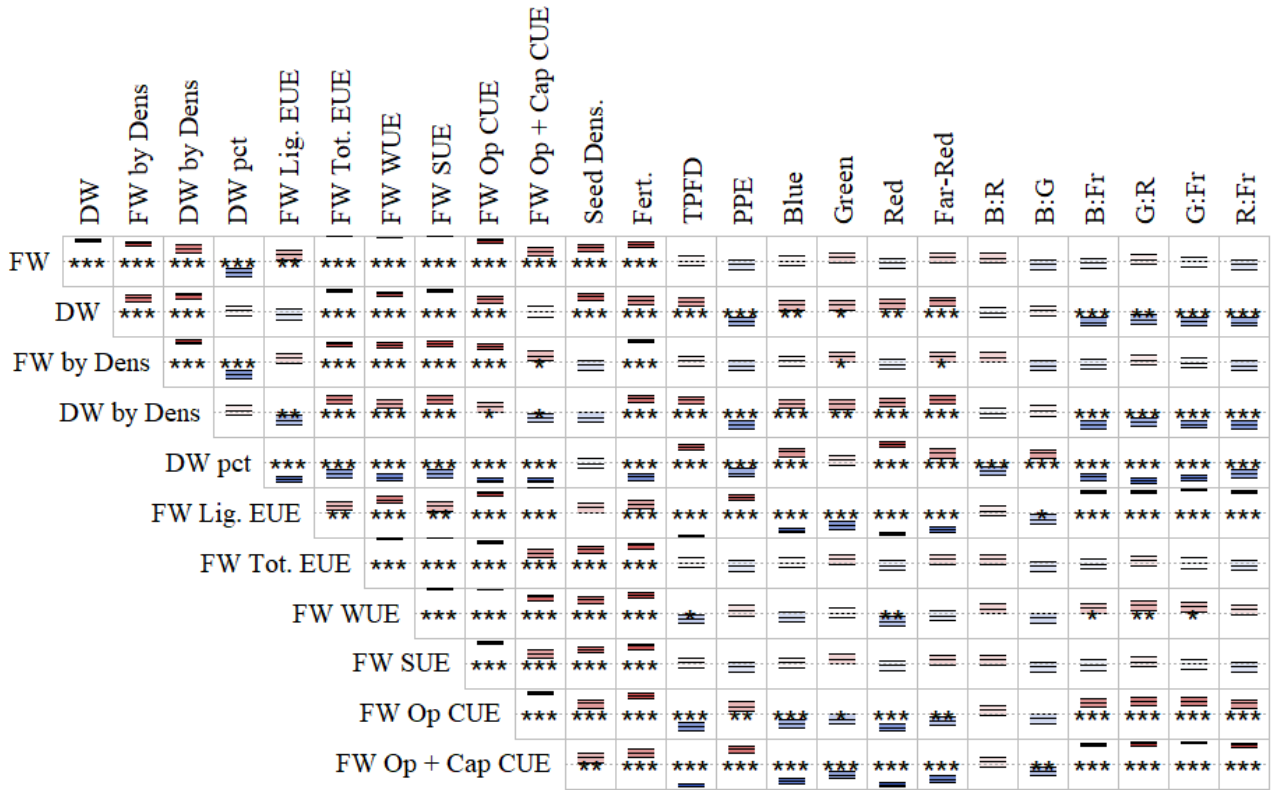

Figure S3. Pearson Correlation with 95% confidence intervals and p-values (α = 0.05; * = p < 0.05, ** = p < 0.01, and *** = p < 0.001) shown for kohlrabi microgreens for the following parameters: FW(kg/m^2), DW (kg/m^2), FW by Density (g FW/g seed), DW by Density (g DW/g seed), DW pct (%), FW Light EUE (g FW/kWh light), FW Total EUE (g FW/kWh total), FW WUE (g FW/L H2O), FW SUE (g FW/m^2/day), FW Op CUE (g FW/dollar operating costs), FW Op + Cap CUE (g FW/dollar operating and capital costs), SD, Fertilizer, Total Photon Flux Density (TPFD), PPE, Blue, Green, Red, and Far-Red light components, B:R, B:G, B:Fr, G:R, G:Fr, and R:Fr ratios. Red indicates positive correlation, up to a perfect score of 1, White indicates no correlation, and Blue indicates negative correlation, down to a perfect score of -1.

1.1.2 Kohlrabi Resource Use Efficiency

Figure S4 below shows the different resource use efficiencies for the production of kohlrabi microgreens. The top row shows the EUE of only the power consumption of the lights considered. The 24VHELED lights are by far the most significantly energy efficient contributors to fresh biomass production. Interestingly, given the lower power consumption of the NG Light Recipe, due to the highly efficient photon conversion efficiency of the Blue and Red band LEDs, this lower performing recipe for biomass had a higher EUE than other OSRAM Light Recipes, although it was only significantly greater than the HBG at High SD with Fertilizer. In general, the HBG recipe was a good producer of biomass, but was energetically expensive, and it was significantly lower for EUE than multiple recipes across treatments. On average, the OSRAM Light Recipes produced around 116.43 g FW/kWh, while the 24VHELED produced around 665.74 g FW/kWh, averaged across densities and Fertilizer. However, light was not the only energy consumer: we therefore separated this EUE (total) from the aforementioned EUE (lights). The Total EUE showed that although there were differences between the light use efficiencies, as they were a small contributing component, the overarching pattern of biomass production is shown here. As such, the HFR recipe performed best, and the 24VHELED performed slightly better due to its EUE advantages. Overall, the four lowest performing Light Recipes produced around 1.32 g FW/kWh, while the HFR produced around 1.58 g FW/kWh. There is a similar lack of transformation of values for WUE

(total evapotranspiration of the system), except that once again, the 24VHELED had a markedly better WUE compared to the OSRAM Light Recipes (consuming around 11.86 liters over 8 days compared to 14.18 liters over 8 days for the OSRAM). However, this gave it a significant advantage only over the NG and HR recipes without Fertilizer, as it was very similar to the HFR. The WUE results overall show that all production methods had similar efficiencies, with Fertilizer contributing much more to improvements to WUE (24.18 g FW/liter H_2O Unfertilized vs. 38.04 g FW/liter H_2O Fertilized). The fourth row down shows the SUE of kohlrabi microgreens, where the patterns of biomass production are evident when comparing treatments. However, it is worth noting that the best performing Light Recipe, the HFR, produced around 185.26 g/m^2/day averaged across treatments, compared to the lowest of 142.55 g/m^2/day for the NG recipe. The fifth row down shows the CUE in g FW/dollar operating costs. For kohlrabi, the HFR and 24VHELED Light Recipes were the best, and were not significantly different, with around 43.72 g FW/dollar and 35.81 g/dollar respectively. This is due to the efficiency advantages of the 24VHELEDs and the production advantages of the HFR. The NG recipe, generally one of the lowest performing recipes for pure biomass production, was more cost efficient in this case because of its energy efficiency. We expanded on this CUE by also including the costs of the lights and other upfront capital inputs costs. We showed that the 24VHELED was by far the most cost-efficient Light Recipe, producing around 0.14 g FW/dollar; the lowest performing recipe was the NG recipe at around 0.04 g FW/dollar. These resource use efficiency figures demonstrate the tradeoffs inherent in microgreen production. Although the higher intensity, programmable OSRAM LEDs performed the best for biomass, there are other dynamics to consider when selecting production methods. For instance, with the OSRAM lights producing on average 1.07 times the FW without Fertilizer, and 1.10 times the biomass without Fertilizer, it would take 427 generations and 299 generations, respectively, to make up for this cost deficit, assuming each cup of microgreens sold for 3 dollars and there were 80 cups (full capacity) per layer. For DW, this is even more pronounced. Perhaps once the efficiency of Green and Far-Red LED bands catches up to Red and Blue bands, and the costs of high-quality LEDs are lowered, the resource use efficiency will change. For now, as is common practice for growers, cheap and easily accessible LEDs produce less, but competitive, amounts of microgreens.

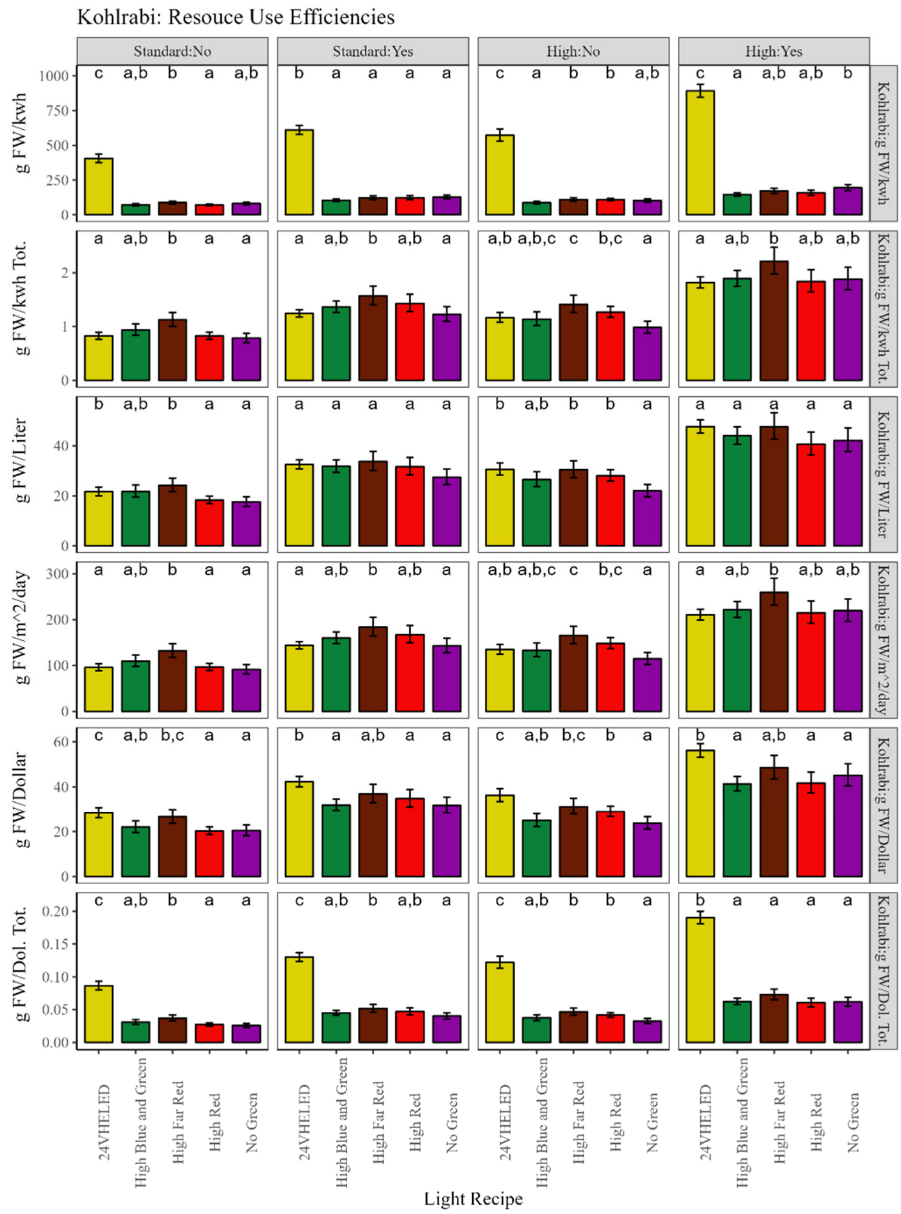

Figure S4. Facet graph detailing the kohlrabi means with 95% confidence intervals at every combination of our experimental factors for the EUE of Lights (top; g FW/kWh), Total EUE (second from top; g FW/kWh), WUE (third from top; g FW/Liter H_2O), SUE (third from bottom; g FW/m^2/day), CUE of operating costs (second from bottom; g FW/dollar), and Total CUE (bottom; g FW/dollar). Lowercase letters show the results of Tukey's HSD within each panel, where common letters are not significantly different from one another at $\alpha = 0.05$.

1.2 Mustard

1.2.1 Mustard Production

The multiway ANOVA results from Table S5 demonstrate that for mustard, there were also significant differences for Fresh Weight biomass production due to Light Recipe, Fertilizer, and SD main effects ($p < 0.001$, $p < 0.001$, $p < 0.001$, respectively). Unlike for kohlrabi, there were no significant higher order interactions, indicating that for mustard, the production of FW biomass was due to the singular influences of our main effects. Fertilizer was the main effect that

explained the most variation for FW biomass accumulation (48.73%), with SD and Light Recipe having 22.40% and 10.09%, respectively. These values are similar to those seen for kohlrabi. For DW, the same significance of our main effects was seen for Light Recipe, Fertilizer, and SD ($p < 0.001$, $p < 0.001$, $p < 0.001$, respectively), with no higher order significant interactions as well. For DW, the SD explained the most variation (52.81%), with Fertilizer explaining only 20.22%, and Light Recipe explaining around 10.67%.

Table S5. Type I ANOVA summary table for mustard microgreen FW (left) and DW (right). Sources of variation are Light Recipe (LR), Fertilizer (Fert.), Seeding Density (SD), and their interaction. The df is degrees of freedom, SS is sum squares, η^2 is eta-squared as the effect size, MS is mean squares, and p is the p-value. Significance set at $\alpha = 0.05$.

Source of Variation	*df*	*SS (FW)*	*η^2 (FW)*	*MS (FW)*	*p (FW)*	*SS (DW)*	*η^2 (DW)*	*MS (DW)*	*p (DW)*
LR	4	9.49	10.09%	2.37	< 0.001	1.90E-02	10.67%	5.00E-03	< 0.001
Fert.	1	45.82	48.73%	45.82	< 0.001	3.60E-02	20.22%	3.60E-02	< 0.001
SD	1	21.06	22.40%	21.06	< 0.001	9.40E-02	52.81%	9.40E-02	< 0.001
LR:Fert.	4	0.26	0.28%	0.07	0.88	1.00E-03	0.56%	0.00E+00	0.49
Fert.:SD	1	0.18	0.19%	0.18	0.37	0.00E+00	0.00%	0.00E+00	0.91
LR:SD	4	0.33	0.36%	0.08	0.82	1.00E-03	0.56%	0.00E+00	0.40
LR:Fert.:SD	4	0.38	0.41%	0.10	0.78	1.00E-03	0.56%	0.00E+00	0.51
Error		16.51	17.56%	0.22		2.60E-02	14.61%	0.00E+00	

Table S6 (FW, top) and Table S7 (DW, bottom) shows the outputs for linear models that used four LED bands as predictive variables for biomass accumulative variation. Overall, Table S6 shows that, for FW, Far-Red light was significantly associated with increased biomass accumulation without Fertilizer for Standard and High SD, and was nearly significant with Fertilizer for Standard and High SD ($p=0.11$ and $p=0.07$, respectively). Red was once again significantly negatively associated with FW biomass without Fertilizer at Standard SD ($p=0.05$). For DW, the impact was more straightforward where Far-Red light, and only Far-Red light, was significantly positively associated with biomass accumulation at every experimental combination (p-values ranging from < 0.001 to 0.04). Interestingly, the FW Far-Red light effect for the No Fertilizer and High SD treatment had the greatest coefficient for both FW and DW, with the second highest being the No Fertilizer and Standard SD treatment with Far-Red light for DW. Overall, these linear models, using only light wavelength bands as model effects for predicting FW and DW biomass, had coefficients of determination (R^2) ranging from 0.24 at the lowest, to a high of 0.77. On average, these models had an average R^2 of 0.52. In general, the No Fertilizer models explained much more variation than the Yes Fertilizer models.

Table 1. Linear model R^2, coefficients, effect size (%), and effect significance (p) for mustard FW at different treatment combinations (Fertilizer, SD, and wavelength band). Wavelength band was defined as Blue (400-500 nm), Green (500-600 nm), Red (600-700 nm), and Far-Red (700-800).

Fertilizer	*SD*	*Effect*	*R^2 of Model*	*Coefficient*	*η^2*	*p*
Yes	Standard	Far-Red	0.25	1.04E-02	9.79%	0.11
Yes	High	Far-Red	0.24	1.93E-02	12.25%	0.07
No	Standard	Red	0.77	-1.51E-03	4.91%	0.05
No	Standard	Far-Red	0.77	1.51E-02	51.15%	< 0.001
No	High	Far-Red	0.58	1.94E-02	39.01%	< 0.01

Table S7. Linear model R^2, coefficients, effect size (%), and effect significance (p) for mustard DW at different treatment combinations (Fertilizer, SD, and wavelength band). Wavelength band was defined as Blue (400-500 nm), Green (500-600 nm), Red (600-700 nm), and Far-Red (700-800).

Fertilizer	*SD*	*Effect*	*R^2 of Model*	*Coefficient*	*η^2*	*p*
Yes	Standard	Far-Red	0.50	5.32E-04	11.41%	0.04
Yes	High	Far-Red	0.28	8.11E-04	16.50%	0.04
No	Standard	Far-Red	0.74	8.58E-04	39.25%	< 0.001
No	High	Far-Red	0.72	1.46E-03	47.34%	< 0.001

The Pearson's Correlation below (Figure S5) shows the correlations between our primary dependent variables of interest, as well as some of our key independent ones, such as light components, ratios, Fertilizer and SD. Figure S5 shows that FW was positively, but not significantly, correlated with Green and Far-Red light components, while Red and Blue were neutral or negative. For DW, only the Far-Red light component was significantly correlated, although there was positive correlation with Green and TPFD, as there was for kohlrabi. For FW relative production (FW by Seeding Density), Green and Far-Red light were positively correlated, but not significantly. For relative DW production, both Green and Far-Red light were significantly correlated. DW percent (pct) had significant positive correlation with TPFD and Red light. For our resource use efficiency parameters, applying Fertilizer or increasing SD significantly positively correlated with outcomes.

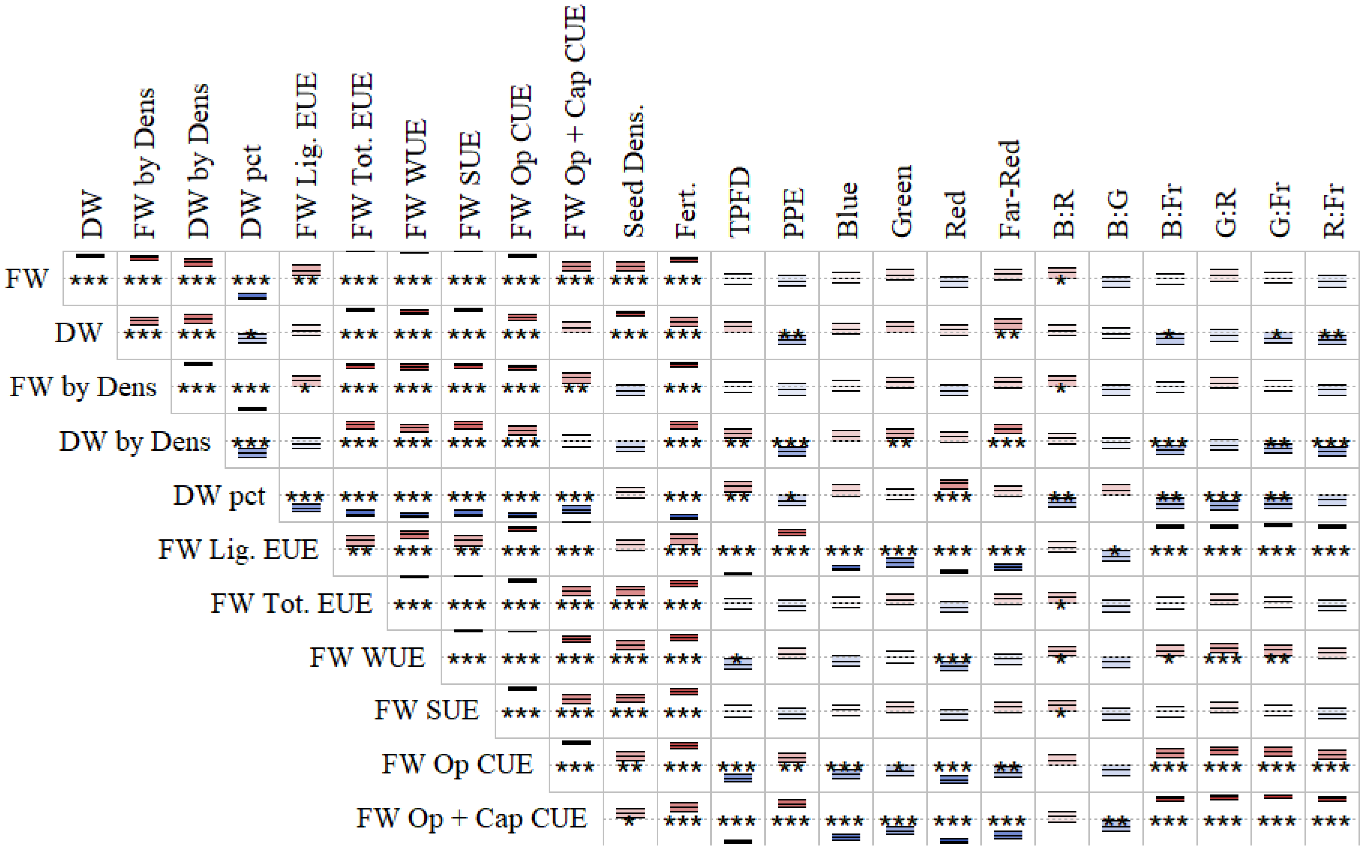

Figure 1. Pearson Correlation with 95% confidence intervals and p-values (α = 0.05; * = $p < 0.05$, ** = $p < 0.01$, and *** = $p < 0.001$) shown for mustard microgreens for the following parameters: FW(kg/m^2), DW (kg/m^2), FW by Density (g FW/g seed), DW by Density (g DW/g seed), DW pct (%), FW Light EUE (g FW/kWh light), FW Total EUE (g FW/kWh total), FW WUE (g FW/L H2O), FW SUE (g FW/m^2/day), FW Op CUE (g FW/dollar operating costs), FW Op + Cap CUE (g FW/dollar operating and capital costs), SD, Fertilizer, Total Photon Flux Density (TPFD), PPE, Blue, Green, Red, and Far-Red light components, B:R, B:G, B:Fr, G:R, G:Fr, and R:Fr ratios. Red indicates positive correlation, up to a perfect score of 1, White indicates no correlation, and Blue indicates negative correlation, down to a perfect score of -1.

1.2.2 Mustard Resource Use Efficiency

Figure S6 below shows the different resource use efficiencies for mustard. The top row shows the EUE of the power consumption of the lights, where the 24VHELED lights were by far the most energy efficient contributors to fresh biomass production. Similar to kohlrabi, the NG Light Recipe had a higher EUE which was significantly the same as HFR and was significantly greater than the HR and HBG in all treatments. Furthermore, in general, the HBG was the second highest producer of biomass, but was energetically expensive, and it was significantly lower than multiple recipes across treatments. On average, the OSRAM Light Recipes produced around 280.24 g FW/kWh, while the 24VHELED produced around 1638.25 g FW/kWh, averaged across Seeding Densities and Fertilizer regime. The Total EUE showed that although there were differences between the light use efficiencies, as they were a small contributing component, the overarching pattern of biomass production is also shown here. As such, the HFR recipe performed best, and the 24VHELED and NG performed slightly better due to their EUE advantages. Overall, the four lowest performing Light Recipes produced around 3.16 g FW/kWh on average across treatments, while the HFR produced around 3.93 g FW/kWh. There was a similar lack of transformation of values for WUE, except that once again, the 24VHELED had a markedly better WUE compared to the OSRAM Light Recipes due to its lower water consumption of 11.86 liters/8 days compared to 14.18 liters/days for the OSRAM lights. This gave it a significant advantage over the NG and HR recipes for all treatments. The WUE results overall show that Fertilizer contributed greatly to the improvements to WUE (56.90 g FW/liter H_2O Unfertilized vs. 92.81 g FW/liter H_2O Fertilized). The fourth row down shows the SUE of mustard microgreens, where the patterns of biomass production are also seen when comparing treatments. For instance, HFR was significantly more space efficient than the other recipes, which produced around 460.06 g/m^2/day, compared to the lowest recipe (HR) at 332.24 g/m^2/day. The fifth row down shows the CUE in g FW/dollar operating costs. For mustard, the 24VHELED and HFR Light Recipes were significantly greater than all three other Light Recipes, with around 107.75 g FW/dollar and 88.88 g/dollar respectively. This is due to the efficiency advantages of the 24VHELEDs and the production advantages of the HFR. We expanded on this CUE by also including the costs of the lights and other upfront capital inputs costs (the grow system and its auxiliary costs) as well. We showed that the 24VHELED was by far the most CUE Light Recipe, with around 0.35 g FW/dollar. The lowest performing recipe was the HR recipe at around 0.09 g FW/dollar. With the OSRAM lights producing on average 1.08 times the FW without Fertilizer, and 1.07 times the biomass without Fertilizer, it would take 373 generations and 427 generations to make up for this deficit, assuming each cup of microgreens sold for 3 dollars and there were 80 cups (full capacity) per layer. This difference is even greater for DW, where the OSRAM lights produced around 1.20 times the DW without Fertilizer, and around 1.11 times the DW biomass with Fertilizer.

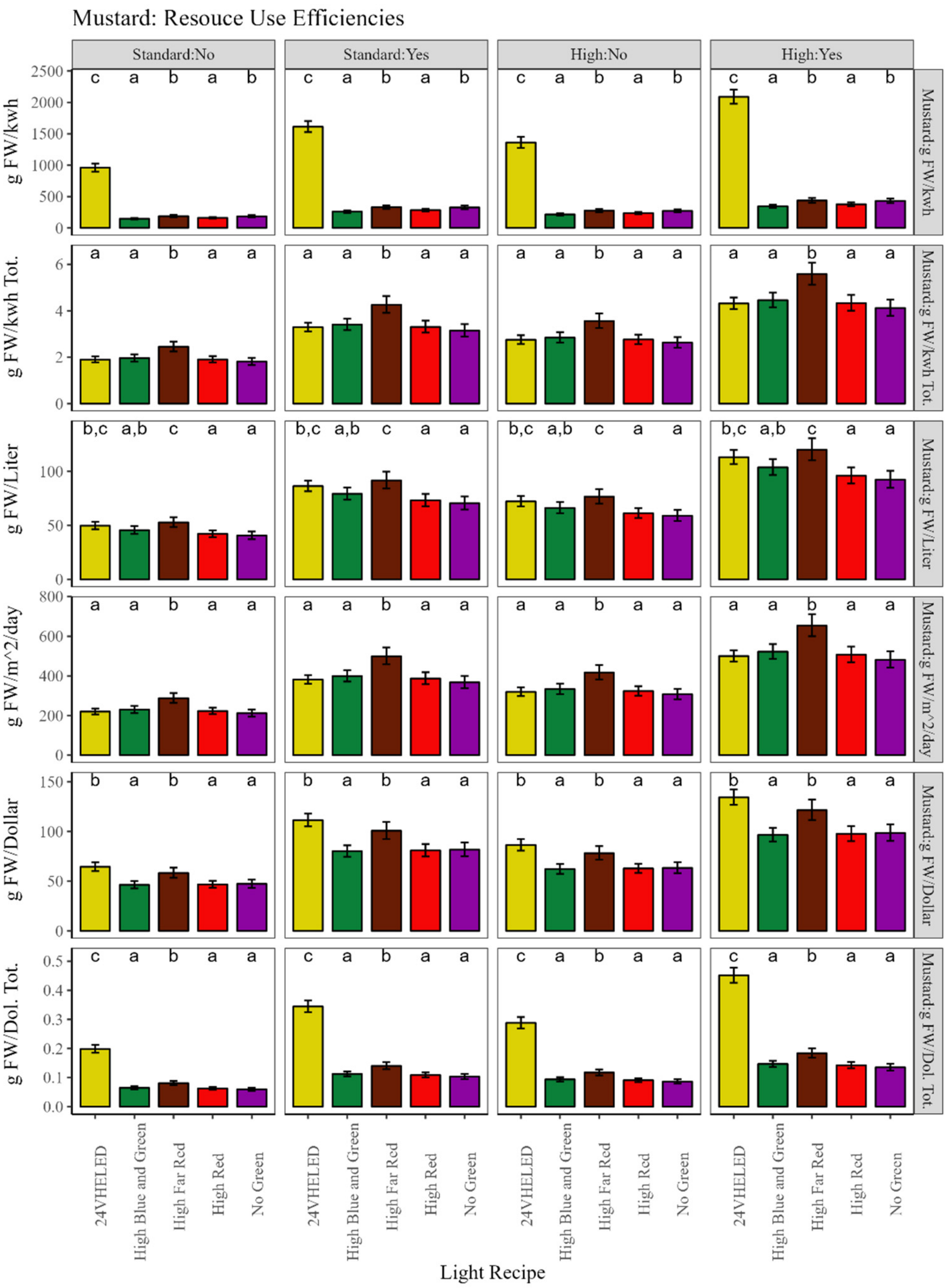

Figure S6. Facet graph detailing the mustard means with 95% confidence intervals at every combination of our experimental factors for the EUE of Lights (top; g FW/kWh), Total EUE (second from top; g FW/kWh), WUE (third from top; g FW/Liter H_2O), SUE (third from bottom; g FW/m^2/day), CUE of operating costs (second from bottom; g FW/dollar), Total CUE (bottom; g FW/dollar). Lowercase letters show the results of Tukey's HSD within each panel, where common letters are not significantly different from one another at $\alpha = 0.05$.

1.3 Radish

1.3.1 Radish Production

The multiway ANOVA results from Table S8 demonstrate that for radish, there were significant differences for Fresh Weight biomass production due to the Light Recipe, Fertilizer, and SD main effects ($p < 0.001$, $p < 0.001$, $p < 0.001$,

respectively). Although it was not significant, similar to kohlrabi, there was a nearly significant interaction between Fertilizer and SD (p=0.07). Once again, Fertilizer explained the most FW variation (50.16%), with SD (27.74%) and Light Recipe (4.88%) having the next highest. For DW, there was also significant differences for Fresh Weight biomass production due to the effects of Light Recipes, Fertilizer, and SD (p < 0.001, p < 0.001, p < 0.001, respectively). And similarly to kohlrabi, the Light Recipe by Fertilizer interaction was significant (p=0.01). For DW, the SD explained the most variation (71.51%), with Light Recipe (12.18%) and SD (6.58%) explaining the next greatest amounts of variation.

Table S8. Type I ANOVA summary table for radish microgreen FW (left) and DW (right). Sources of variation are Light Recipe (LR), Fertilizer (Fert.), Seeding Density (SD), and their interaction. The df is degrees of freedom, SS is sum squares, η^2 is eta-squared as the effect size, MS is mean squares, and p is the p-value. Significance set at $\alpha = 0.05$.

Source of Variation	*df*	*SS (FW)*	*η^2 (FW)*	*MS (FW)*	*p (FW)*	*SS (DW)*	*η^2 (DW)*	*MS (DW)*	*p (DW)*
LR	4	9.31	4.88%	2.33	< 0.001	4.83E-02	12.18%	1.21E-02	< 0.001
Fert.	1	95.68	50.16%	95.68	< 0.001	2.61E-02	6.58%	2.61E-02	< 0.001
SD	1	52.91	27.74%	52.91	< 0.001	2.83E-01	71.51%	2.83E-01	< 0.001
LR:Fert.	4	0.33	0.17%	0.08	0.94	5.88E-03	1.48%	1.47E-03	0.01
Fert.:SD	1	1.35	0.71%	1.35	0.07	7.95E-04	0.20%	7.95E-04	0.15
LR:SD	4	0.35	0.18%	0.09	0.93	1.06E-03	0.27%	2.64E-04	0.60
LR:Fert.:SD	4	0.66	0.35%	0.17	0.80	1.86E-03	0.47%	4.65E-04	0.31
Error		30.15	15.81%	0.40		2.90E-02	7.31%	3.81E-04	

Table S9 (FW, top) and Table S10 (DW, bottom) expands on the relationship between light components and biomass production by showing the R^2, model estimate coefficient, effect size (η^2), and p-value (p) at each treatment level. The linear models used four LED wavelength bands as predictive variables for biomass accumulative variation. Overall, it shows that, for FW, Far-Red light was positively significantly associated with FW accumulation at all levels of experimental factor combinations, except for with Fertilizer at Standard SD, where p=0.06. At the No Fertilizer and Standard SD level, Red light was also found to be a significant predictor, but it had a very small coefficient estimate compared to the Far-Red values. For the No Fertilizer and Standard SD level, Blue was also found to be negatively associated with FW biomass (p=0.07), as it was also for kohlrabi. For DW, it was Red light that was positively, significantly associated with DW production for every combination of experimental factors. Without fertilization, the DW also had Far-Red light as significant positive predictors of DW biomass production at both Density levels; however, this was not seen with Fertilizer for DW. Overall, these linear models, using only light parameters as inputs for predicting FW and DW biomass, had coefficients of determination (R^2) ranging from 0.28 at the lowest, to a high of 0.85. On average, these models had an average R^2 of 0.62. In general, the No Fertilizer models explained much more variation than the Yes Fertilizer models.

Table S9. Linear model R^2, coefficients, effect size (%), and effect significance (p) for radish FW at different treatment combinations (Fertilizer, SD, and wavelength band). Wavelength band was defined as Blue (400-500 nm), Green (500-600 nm), Red (600-700 nm), and Far-Red (700-800).

Fertilizer	*SD*	*Effect*	*R^2 of Model*	*Coefficient*	*η^2*	*p*
Yes	Standard	Far-Red	0.28	1.78E-02	10.89%	0.06
Yes	High	Far-Red	0.29	2.65E-02	12.12%	0.05
No	Standard	Blue	0.85	-4.13E-03	9.62%	0.07
No	Standard	Red	0.85	2.64E-03	9.04%	0.03
No	Standard	Far-Red	0.85	1.54E-02	28.79%	< 0.001
No	High	Far-Red	0.82	1.94E-02	31.45%	< 0.001

Table S10. Linear model R^2, coefficients, effect size (%), and effect significance (p) for radish DW at different treatment combinations (Fertilizer, SD, and wavelength band). Wavelength band was defined as Blue (400-500 nm), Green (500-600 nm), Red (600-700 nm), and Far-Red (700-800).

Fertilizer	*SD*	*Effect*	*R^2 of Model*	*Coefficient*	*η^2*	*p*
Yes	Standard	Red	0.54	2.89E-04	17.84%	< 0.01
Yes	High	Red	0.49	2.73E-04	12.41%	0.03
No	Standard	Red	0.85	3.93E-04	36.88%	< 0.001
No	Standard	Far-Red	0.85	5.25E-04	7.58%	0.02
No	High	Red	0.81	6.19E-04	34.09%	< 0.001
No	High	Far-Red	0.81	1.05E-03	11.17%	0.02

The Pearson's Correlation below (Figure S7) shows the correlations between our primary dependent variables of interest, as well as some of our key independent ones, such as light components, ratios, Fertilizer and SD. Figure S7 shows that FW was positively and significantly correlated with Far-Red light, and was positively, but not significantly, associated with Blue, Green, and Red light. For DW, Blue, Red, and Far-Red light components were significantly correlated, as was TPFD. For FW relative production (FW by Seeding Density), all light components were correlated, but this was mostly significant for relative DW. For our resource use efficiency parameters, applying Fertilizer or increasing SD significantly positively and significantly correlated with outcomes.

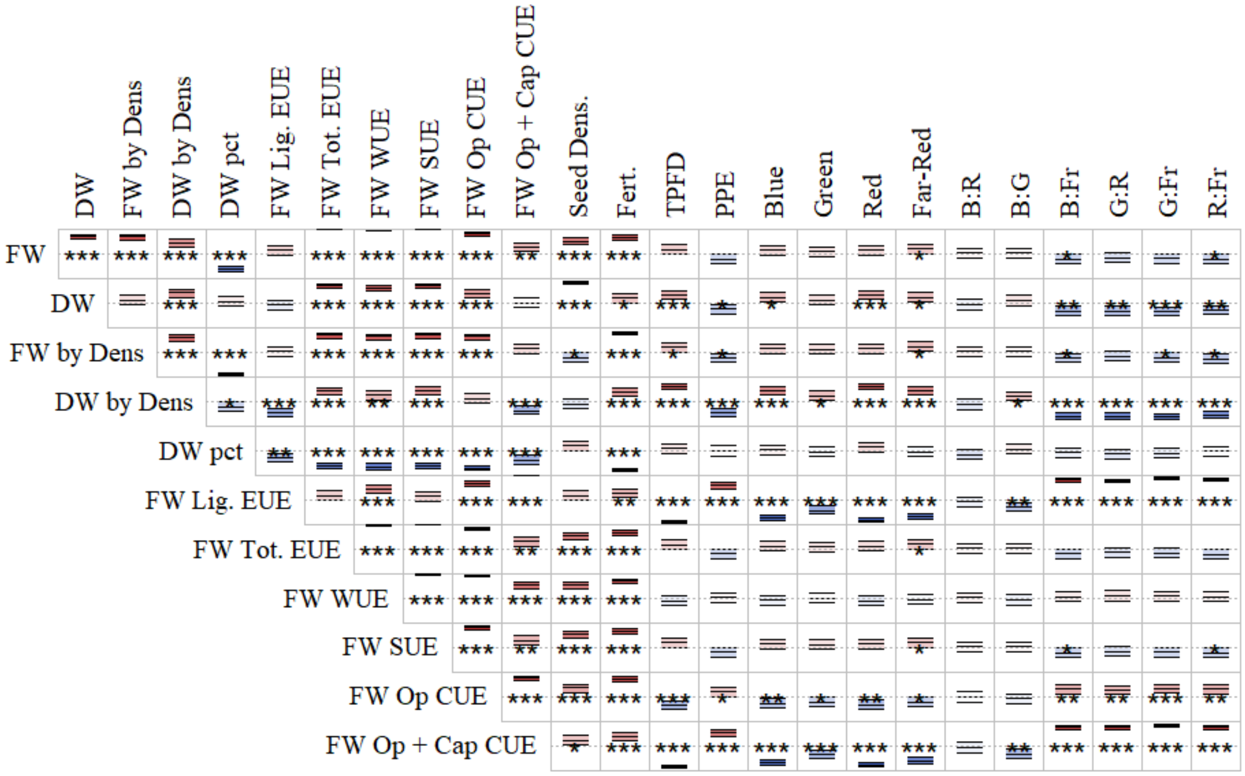

Figure S7. Pearson Correlation with 95% confidence intervals and p-values (α = 0.05; * = $p < 0.05$, ** = $p < 0.01$, and *** = $p < 0.001$) shown for radish microgreens for the following parameters: FW(kg/m^2), DW (kg/m^2), FW by Density (g FW/g seed), DW by Density (g DW/g seed), DW pct (%), FW Light EUE (g FW/kWh light), FW Total EUE (g FW/kWh total), FW WUE (g FW/L H2O), FW SUE (g FW/m^2/day), FW Op CUE (g FW/dollar operating costs), FW Op + Cap CUE (g FW/dollar operating and capital costs), SD, Fertilizer, Total Photon Flux Density (TPFD), PPE, Blue, Green, Red, and Far-Red light components, B:R, B:G, B:Fr, G:R, G:Fr, and R:Fr ratios. Red indicates positive correlation, up to a perfect score of 1, White indicates no correlation, and Blue indicates negative correlation, down to a perfect score of -1.

1.3.2 Radish Resource Use Efficiency

Figure S8 below shows the different resource use efficiencies for radish. The top row shows the EUE with the power consumption of the lights, where the 24VHELED lights were by far the most significantly energy efficient. Once again, the NG Light Recipe had a higher EUE and was significantly greater than the HFR, HR, and HBG recipes in all treatments. Furthermore, the HBG recipe was significantly lower than all other Light Recipes at all treatment levels. On average, the OSRAM Light Recipes produced around 474.76 g FW/kWh light, while the 24VHELED produced around 2466.98 g FW/kWh light, averaged across densities and Fertilizer. The Total EUE showed that although there were differences between the light use efficiencies, because they were a small contributing component of the overall energy use, the overarching pattern of biomass production was also shown here. In contrast to both the kohlrabi and the mustard, where the 24VHELED made improvements that brought it into line with the other recipes concerning EUE total, even though it was lower for total biomass production, for radish the 24VHELED was significantly lower than all other Light Recipes at all treatment levels. The highest EUE total was seen with the HFR Light Recipe, with a value of 5.95 g FW/kWh; the lowest EUE total was 24VHELED at 4.99 g FW/kWh. In the third row, we have shown the WUE in g FW/Liter H_2O. There was a similar lack of transformation of relationships for WUE, except that once again, the 24VHELED had a better WUE compared to the OSRAM Light Recipes due to its lower water consumption. The HFR had the highest WUE, with the NG recipe always significantly less than the HFR at all treatment levels. The WUE results overall show that Fertilizer contributed greatly to the improvements to WUE (94.06 g FW/liter H_2O Unfertilized vs. 146.10 g FW/liter H_2O Fertilized). The fourth row down shows the SUE of radish microgreens, where the HFR recipe was significantly more space efficient than the other recipes. It is worth noting that the best performing Light Recipe, HFR, produced around 697.06 g/m^2/day, compared to 577.94 g/m^2/day for the lowest recipe, 24VHELED. The fifth row down shows the CUE in g FW/dollar operating costs. For radish, the 24VHELED Light Recipe was significantly greater than the HBG, HR, and NG recipes at all treatment levels; only the HFR was comparable, which was only significantly greater than the HBG at all treatment levels. The 24VHELED had around 148.70 g FW/dollar and 127.41 g/dollar for the HFR recipe, while the HBG was the lowest. We expanded on this CUE by also including the costs of the lights and other capital inputs costs (the grow system and its auxiliary material costs), shown in the bottom row. We showed that the 24VHELED was by far the most cost-efficient Light Recipe, with around 0.52 g FW/dollar, which was significantly greater than all other Light Recipes, while the HFR recipe was significantly higher than the HBG and the NG. The lowest performing recipes were around 0.18 g FW/dollar. With the OSRAM lights producing on average 1.22 times the FW without Fertilizer, and 1.13 times the biomass without Fertilizer, it would take 136 generations and 230 generations, respectively, to make up for this deficit, assuming each cup of microgreens sold for 3 dollars and there were 80 cups (full capacity) per layer. This would be even greater for DW, as the OSRAM produced around 1.29 times the DW biomass without Fertilizer, and around 1.13 times the DW biomass with Fertilizer.

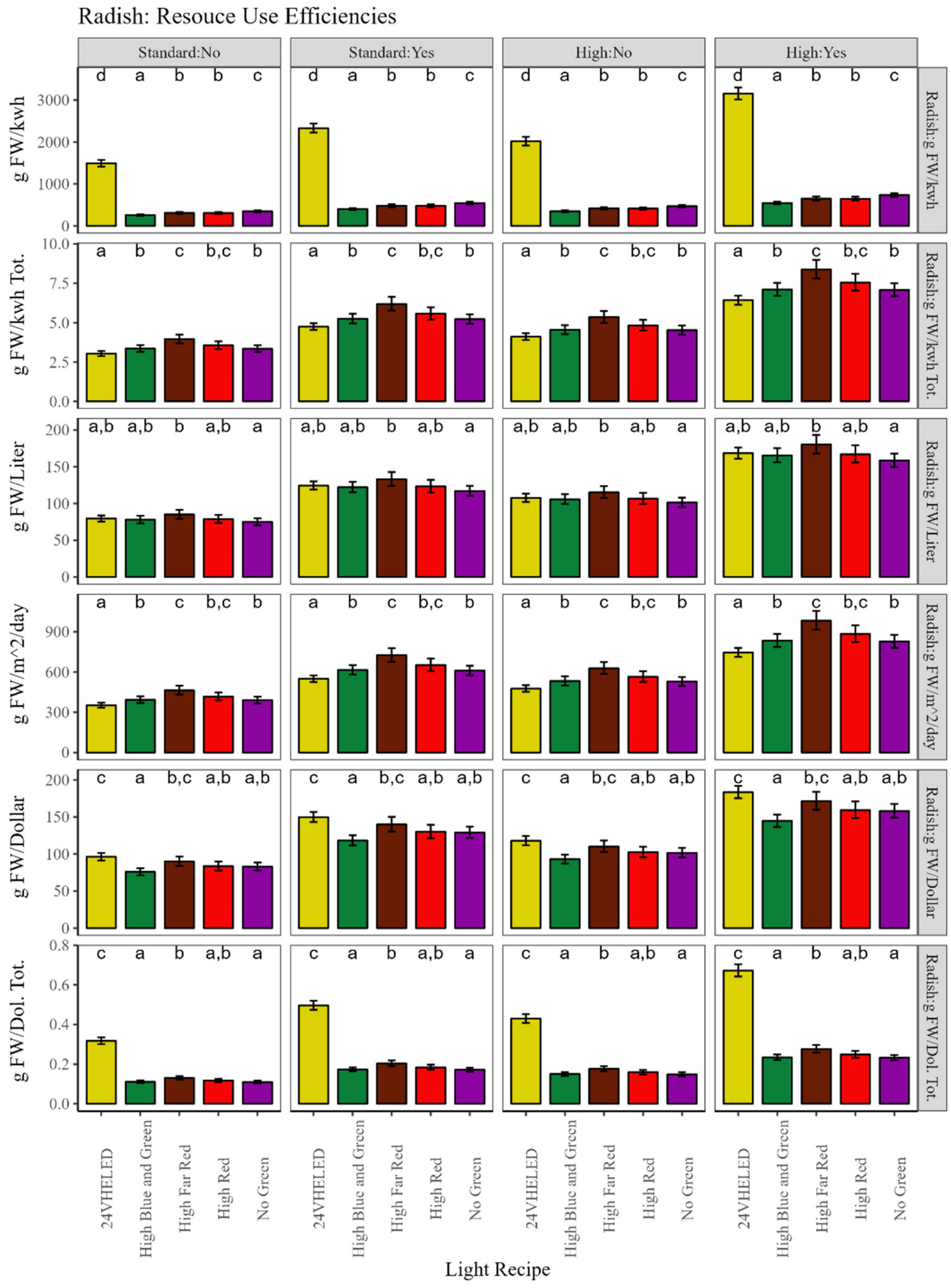

Figure S8. Facet graph detailing the radish means with 95% confidence intervals at every combination of our experimental factors for the EUE of Lights (top; g FW/kWh), Total EUE (second from top; g FW/kWh), WUE (third from top; g FW/Liter H_2O), SUE (third from bottom; g FW/m^2/day), CUE of operating costs (second from bottom; g FW/dollar), Total CUE (bottom; g FW/dollar). Lowercase letters show the results of Tukey's HSD within each panel, where common letters are not significantly different from one another at $\alpha = 0.05$.
